# Family Environment Variables as Predictors of School Absenteeism Severity at Multiple Levels: Ensemble and Classification and Regression Tree Analysis

**DOI:** 10.3389/fpsyg.2019.02381

**Published:** 2019-10-18

**Authors:** Mirae J. Fornander, Christopher A. Kearney

**Affiliations:** University of Nevada, Las Vegas, Las Vegas, NV, United States

**Keywords:** absenteeism severity, truancy, ensemble analysis, classification and regression tree analysis, family environment, risk variables

## Abstract

School attendance problems, including school absenteeism, are common to many students worldwide, and frameworks to better understand these heterogeneous students include multiple classes or tiers of intertwined risk factors as well as interventions. Recent studies have thus examined risk factors at varying levels of absenteeism severity to demarcate distinctions among these tiers. Prior studies in this regard have focused more on demographic and academic variables and less on family environment risk factors that are endemic to this population. The present study utilized ensemble and classification and regression tree analysis to identify potential family environment risk factors among youth (i.e., children and adolescents) at different levels of school absenteeism severity (i.e., 1 + %, 3 + %, 5 + %, 10 + %). Higher levels of absenteeism were also examined on an exploratory basis. Participants included 341 youth aged 5–17 years (*M* = 12.2; SD = 3.3) and their families from an outpatient therapy clinic (68.3%) and community (31.7%) setting, the latter from a family court and truancy diversion program cohort. Family environment risk factors tended to be more circumscribed and informative at higher levels of absenteeism, with greater diversity at lower levels. Higher levels of absenteeism appear more closely related to lower achievement orientation, active-recreational orientation, cohesion, and expressiveness, though several nuanced results were found as well. Absenteeism severity levels of 10–15% may be associated more with qualitative changes in family functioning. These data may support a Tier 2-Tier 3 distinction in this regard and may indicate the need for specific family-based intervention goals at higher levels of absenteeism severity.

## Introduction

School attendance problems, including school absenteeism, are common to many students worldwide (UNESCO, 2012). School absenteeism has been linked to academic performance and achievement deficiencies, various mental health and social problems, and later school dropout (Bridgeland et al., 2006; Burton et al., 2014; Attwood and Croll, 2015). School attendance problems leading to dropout can have lingering effects into adulthood as well, including increased risk for eventual economic, marital, occupational, and psychiatric problems (Christenson and Thurlow, 2004; Rocque et al., 2017; Mazerolle et al., 2018).

Recent theoretical frameworks of school attendance problems have focused on multiple classes or tiers of intertwined risk factors as well as interventions to fully capture the complexity of this heterogeneous population (Kearney, 2008; Kearney and Graczyk, 2014; Skedgell and Kearney, 2018; Ingul et al., 2019). Researchers have identified general classes of factors, such as child, parent, family, peer, school, and community variables, which enhance risk for school attendance problems (Ready, 2010; Burrus and Roberts, 2012; Ingul et al., 2012; Havik et al., 2015; Maxwell, 2016; McKee and Caldarella, 2016). These classes of risk factors often work in tandem, particularly with respect to chronic and severe school attendance problems and school dropout (Freeman and Simonsen, 2015).

Family environment type may be one such risk factor that directly impacts school attendance and academic achievement in youth (Epstein and Sheldon, 2002; Hill and Taylor, 2004). Bernstein et al. (1990, 1999) and Bernstein and Borchardt, 1996, for example, identified several family variables associated with anxiety-based school refusal. These variables included lack of agreement among family members with respect to roles, inconsistency of family rules, and greater communication difficulties, rigidity, and disengagement. Lagana (2004) found that low family cohesion was more characteristic of students at medium to high risk of school dropout than those at low risk. Family structure and culture relate closely to school dropout as well (De Witte et al., 2013).

Kearney and Silverman (1995) identified various dynamic subtypes among families of youth with broader school refusal behavior: enmeshed, detached, isolated, conflictive, healthy, and mixed. Enmeshed families display extreme closeness, emotional dependency, over-involvement, and loyalty but lack developmentally appropriate autonomy, leading some youth to feel insecure and display internalizing and externalizing symptoms (Barber and Buehler, 1996; Davies et al., 2004; Berryhill et al., 2018). Detached family members are relatively uninvolved or inattentive to one another, leading some youth to display internalizing and externalizing symptoms, poor emotional regulation, and insecure relationships with family members (Weiss and Cain, 1964; Davies et al., 2004; Lindblom et al., 2017).

Conflictive families display a lack of intimacy and emotional expression in addition to high rates of struggle and hostility among family members, leading some youth to display internalizing symptoms and risk-taking behaviors (Makihara et al., 1985; Jaycox and Repetti, 1993; Bradley et al., 2010; Chen et al., 2017). Isolated families are characterized by minimal, if any, contact with people outside of the family, leading some youth to experience stress and social withdrawal (Wahler, 1980; Tucker and Rodriguez, 2014). Healthy families are characterized by adaptive functioning and good communication and problem-solving skills. Mixed families display characteristics of several of these patterns (Kearney and Silverman, 1995; Barber and Buehler, 1996).

In addition, researchers have begun to focus on the concept of multi-tiered systems of support (MTSS) and related models to conceptualize different layers of intervention for school attendance problems (Freeman et al., 2016; Kearney, 2016; Elliott and Place, 2019). MTSS aims to provide high-quality, individualized instruction, and intervention, informed by frequent progress monitoring, for all aspects of student education (McIntosh and Goodman, 2016). MTSS models are often arranged in three tiers that focus on prevention (Tier 1), early intervention for emerging, acute problems (Tier 2), and intensive intervention for chronic and severe problems (Tier 3; Eagle et al., 2015). MTSS models have been applied to academic, social, and behavioral problems and skills across various age ranges and school settings (August et al., 2018).

Kearney and Graczyk (2014) were the first to apply MTSS principles to a model of school absenteeism directly. Each MTSS tier has a specific focus based on the severity of school absenteeism: (1) Tier 1 focuses on enhancing functioning and schoolwide attendance and preventing absenteeism for all students, (2) Tier 2 focuses on addressing students with emerging, acute, or mild to moderate school absenteeism, and (3) Tier 3 focuses on addressing students with chronic and severe school absenteeism (Kearney, 2016). Specific interventions are matched to each tier to help school personnel identify individualized responses. Recent research has demonstrated the value of applying MTSS models to school absenteeism. For example, schools that implement MTSS with higher fidelity have lower levels of school absenteeism than schools with less fidelity (Freeman et al., 2016). School districts may also include attendance measures in MTSS models (Coffey et al., 2018).

A key task for researchers utilizing MTSS models for school absenteeism has been to identify demarcations between the tiers. A distinction between Tiers 1 and 2 essentially means a distinction between nonproblematic and problematic behavior, such as between appropriate school attendance and school absenteeism in need of intervention (Pullen and Kennedy, 2019). However, no consistent, consensus definition for problematic school absenteeism exists across research disciplines or school districts (Gentle-Genitty et al., 2015; Spruyt et al., 2016). Greater consensus can be found with respect to distinguishing Tiers 2 and 3, or identifying at what point school absenteeism is chronic and severe (DePaoli et al., 2015). Researchers, school districts, and other agencies sometimes utilize a 10% absenteeism cutoff to identify chronic absenteeism, though this is somewhat arbitrary and not universal (Conry and Richards, 2018).

Specific data-based demarcations between these tiers remain sparse, despite the fact that such distinctions would help inform early warning systems and intervention assignments for student absenteeism (Chu et al., 2018). Skedgell and Kearney (2016, 2018) found that risk factors for levels of absenteeism at 10% or higher tended to be more restricted than risk factors at lower levels of absenteeism. These studies focused primarily on academic and demographic variables, however, without examining family factors that have been identified as a key correlate of school attendance problems (Dahl, 2016).

The present study aimed to identify potential family environment risk factors among youth at different levels of school absenteeism severity (i.e., 1 + %, 3 + %, 5 + %, 10 + %). Participants included students referred for services due to substantial school absenteeism, which allowed for analysis of varying levels of severity. In accordance with recent calls to employ machine learning-based methods to examine risk factors for school absenteeism (Chung and Lee, 2019; Sansone, 2019), two sets of statistical approaches were utilized. Ensemble analysis, including chi-square adjusted interaction detection (CHAID), support vector machines, and neural network analyses, is a nonparametric method that combines multiple algorithmic models or classifiers to produce a single best model for a given data set (Berk, 2006). In addition, classification and regression tree analysis (CART) is a nonparametric method that identifies comprehensive subgroups based on interactions among multiple risk or predictor variables (Lemon et al., 2003). Nonparametric methods are increasingly used for academic variables denoted by categorical levels (e.g., Cordero et al., 2017; Lahti et al., 2019). Various levels of school absenteeism were examined, with a general expectation that risk factors at higher levels of absenteeism would be more restricted than risk factors at lower levels of absenteeism.

## Materials and Methods

### Participants

Participants included 341 youth (i.e., children and adolescents) aged 5–17 years (*M* = 12.2; SD = 3.3) and their families from an outpatient therapy clinic (68.3%) and community (31.7%) setting, the latter from a family court and truancy diversion program cohort. For the clinic sample, age range was 5–16 years (*M* = 11.0; SD = 3.2). Participants were primarily male (62.9%) and were European-American (78.2%), Asian (11.6%), Hispanic (5.8%), African American (2.2%), multiracial or biracial (1.3%), and other (0.4%). For the community sample, age range was 11–17 years (*M* = 14.8; SD = 1.5). Participants were primarily female (53.7%) and were Hispanic (75.0%), African American (10.2%), other (5.6%), multiracial or biracial (3.7%), Asian (2.8%), and European-American (2.8%). Across both groups, most parents were married (50.0%); others were divorced (17.1%), separated (16.7%), never married (15.2%), or had another status (1.0%). Most fathers (57.0%) and mothers (63.3%) had graduated high school. Participants missed an average of 19.0% days of school (SD = 17.2) at time of assessment. Some youths were referred for treatment for school refusal behaviors (e.g., distress at school, morning misbehaviors designed to miss school, skipped classes, and tardiness) that did not include formal full-day absences.

### Measures

The Family Environment Scale: Form R (FES; Moos and Moos, 2009) is a 90-item true/false measure of current family relationships, personal growth, and family system maintenance. The FES comprises 10 subscales based on standard scores (mean, 50): cohesion (family member support of one another; COH), expressiveness (encouraging expression of feelings; EXP), conflict (open anger and hostility; CON), independence (self-sufficient, assertive members; IND), achievement orientation (activities cast in a competitive framework; ACH), intellectual-cultural orientation (family interest in intellectual and cultural issues; ICO), active-recreational orientation (participation in recreational/social activities; ARO), moral-religious emphasis (emphasis on ethical and religious values; MRE), organization (clear structure in activities; ORG), and control (set rules and procedures to structure family life; CTL). Internal consistency (Cronbach’s alpha) ranges between 0.61 and 0.78. Cronbach’s alpha for the items in the present study was 0.72. Two- and four-month test-retest reliabilities range between 0.70 and 0.91 (Moos, 1990). FES item and subscale standard scores (*M* = 50.0) were utilized as the primary unit of analysis in the present study.

School staff or parents provided absenteeism severity data in the form of number of full school days missed. Percentage of full school days missed was calculated by dividing a student’s total number of full school days missed by the number of days of school in that academic year, at the time of assessment, and then multiplying that number by 100.

### Procedure and Data Analyses

Participants were recruited from a specialized outpatient therapy clinic or community setting. Participants in the community setting were referred to family court or a truancy diversion program by their school or parent(s)/guardian(s) based on prior school absences. Measures that included the FES were administered to youth and their parent(s)/guardian(s) independently and in the presence of a research assistant. Spanish versions of the measures were available. Study procedures, including parent consent and child assent, were approved by a university institutional review board.

Ensemble analysis was utilized to identify potential family environment risk factors among youth with school attendance problems across different levels of school absenteeism. Ensemble analysis is the combination of multiple algorithmic models or classifiers to produce one, best model that can be applied to the data (Berk, 2006). These models have been shown to outperform standard parametric methods, primarily due to the automation of identifying interactions and non-linearities and reducing overestimations of a model’s predictive ability (Rosellini et al., 2018). Ensemble analysis can include many different statistical methods; the present study utilized chi-square adjusted interaction detection (CHAID) decision trees, support vector machines, and neural network analyses. Predictors were examined collectively and independently. A multiple imputation method was utilized; different plausible imputed data sets were examined, and combined results were obtained and reported here. Confusion matrices supported the use of CHAID decision trees as the best approach. In addition, CART analyses were utilized to more specifically examine clusters of FES items associated with enhanced risk for a particular level of absenteeism severity (i.e., 1 + %, 3 + %, 5 + %, 10 + %). Other absenteeism levels were examined on an exploratory basis (i.e., 15 + %, 20 + %, 30 + %, 40 + %). For brevity, significant results are reported.

## Results

### Absenteeism: 1 + %

For the CHAID analysis, the final collective tree-model that best differentiated youth with 1 + % absenteeism from youth with <1% absenteeism correctly identified 99.4% of participants and identified two main risk factors: FES items 1 and 44. Youth with items 1 (members help and support one another; COH) and 44 (little privacy in our family; IND) endorsed as true were at higher risk for 1 + % absenteeism (66.5%); youth with items 1 and 44 endorsed as false were at lower risk (27.6%). The tree-model demonstrated higher sensitivity than specificity. Independent analysis of the predictors revealed that ARO scores significantly predicted 1 + % absenteeism (*p* < 0.02, *F* = 9.58). ARO scores of <=53.0 indicated higher risk for 1 + % absenteeism (80.1%); ARO scores of >53.0 indicated lower risk (19.9%). IND scores also significantly predicted 1 + % absenteeism (*p* < 0.05, *F* = 7.39). IND scores of >37.0 indicated higher risk for 1 + % absenteeism (67.7%); IND scores of <=37.0 indicated lower risk (32.3%).

CART item analysis identified three subgroups at highest risk for 1 + % absenteeism (each node at 100.0%): (1) items 28 (true; talk about religious meaning; MRE) and 40 (true; set ways of doing things; CTL); (2) items 28 (true; talk about religious meaning; MRE), 39 (true; on time is very important; ORG), 40 (false; set ways of doing things; CTL), and 62 (true; money/bills openly talked about; EXP); and (3) items 28 (false; talk about religious meaning; MRE), 29 (true; hard to find things; ORG), and 44 (true; very little privacy in family; IND). The tree-model’s accuracy in predicting 1 + % absenteeism was approximately 91.3%.

### Absenteeism: 3 + %

For the CHAID analysis, the final collective tree-model that best differentiated youth with 3 + % absenteeism from youth with <3% absenteeism correctly identified 83.2% of participants and identified several items (2, 25, 31, 42, 62, and 89) and subscale scores as risk factors (Table 1). The tree-model demonstrated higher sensitivity than specificity. The final node representing highest overall risk of 3 + % absenteeism (0.968) included items 2 (true; members keep feelings to self; EXP), 25 (true), and 42 (true; doing things spur of the moment; EXP). Independent analysis of the predictors revealed that ARO scores significantly predicted 3 + % absenteeism (*p* < 0.01, *F* = 12.62). ARO scores of <=53.0 indicated higher risk for 3 + % absenteeism (80.1%); ARO scores of >53.0 indicated lower risk (19.9%).

**Table 1:** FES subscale standard scores predictive of 3 + % absenteeism.

	Higher risk	Lower risk
Expressiveness	34.0–51.5 (8.6%)	59.0–60.0 (3.2%)
Achievement orientation	>47.0 (4.3%)	<=47.0 (4.2%)
Moral-religious emphasis	<=61.0 (5.0%)	>61.0 (2.7%)
Independence	<=37.0 (2.4%)	>37.0 (2.3%)

*Subscales presented in descending order of impact*.

CART item analysis identified four subgroups at highest risk for 3 + % absenteeism (each node at 100.0%): (1) items 25 (true; money not very important to us; ACH) and 31 (true; feeling of family togetherness; COH); (2) items 25 (false; money not very important to us; ACH), 31 (false; feeling of family togetherness; COH), and 89 (true; dishes done immediately after eating; ORG); (3) items 2 (true; members keep feelings to self; EXP), 5 (true; important to be best; ACO), 25 (true; money not very important to us; ACH), and 53 (false; members sometimes hit; CON); and 4) items 2 (false; members keep feelings to self; EXP), 14 (false; encouraged to be independent; IND), 25 (true; money not very important to us; ACH), 86 (true; like art and music; ICO), and 90 (false; cannot get away with much; CTL). The tree-model’s accuracy in predicting 3 + % absenteeism was approximately 85.7%.

### Absenteeism: 5 + %

For the CHAID analysis, the final collective tree-model that best differentiated youth with 5 + % absenteeism from youth with <5% absenteeism correctly identified 76.3% of participants and identified several items (2, 29, 35, 40, 50, 62, and 71) and subscale scores as risk factors (Table 2). The tree-model demonstrated higher sensitivity than specificity. The final node representing highest overall risk of 5 + % absenteeism (0.986) included items 2 and 29 (true) and IND scores of <=37. Independent analysis of the predictors revealed that ARO scores significantly predicted 5 + % absenteeism (*p* < 0.02, *F* = 9.57, predicted 0.760). ARO scores of <=53.0 indicated higher risk for 3 + % absenteeism (80.1%); ARO scores of >53.0 indicated lower risk (19.9%).

**Table 2:** FES subscale standard scores predictive of 5 + % absenteeism.

	Higher risk	Lower risk
Expressiveness	40.8–51.5 (10.0%)	59.0–60.0 (3.7%)
Cohesion	>32.7 (10.2%)	<=32.7 (3.1%)
Independence	>37.0 (4.9%)	<=37.0 (3.0%)
Moral-religious emphasis	<=61.0 (3.5%)	>61.0 (2.3%)
Conflict	>43.0 (7.8%)	<=43.0 (2.2%)

*Subscales presented in descending order of impact*.

CART item analysis identified three subgroups at highest risk for 5 + % absenteeism (each node at 100.0%): (1) items 51 (true; members back each other; COH), 56 (false; someone plays a musical instrument; ICO), and 77 (true; members go out a lot; ARO); (2) items 34 (false; we come and go as we want; IND), 45 (true; strive to do things better; ACO), 74 (true; hard to be by self without hurting feelings; IND), and 77 (false; members go out a lot; ARO); and (3) items 16 (true; rarely go to plays/concerts; ICO), 17 (false; friends often come over; ARO), 29 (false; hard to find things; ORG), 74 (false; hard to be by self without hurting feelings; IND), and 77 (false; members go out a lot; ARO). The tree-model’s accuracy in predicting 5 + % absenteeism was approximately 74.5%.

### Absenteeism: 10 + %

For the CHAID analysis, the final collective tree-model that best differentiated youth with 10 + % absenteeism from youth with <10% absenteeism correctly identified 58.3% of participants and identified several items (4, 11, 16, 17, 44, 49, 68, 79, and 87) and subscale scores as risk factors (Table 3). The tree-model demonstrated higher sensitivity than specificity. The final node representing highest overall risk of 10 + % absenteeism (1.000) included ORG scores of 53.0–58.0, ICO scores of 35.9–41.0, and item 17 (true; friends come over; ARO). Independent analysis of the predictors revealed that COH scores significantly predicted 10 + % of days missed. COH scores of <=52.0 indicated higher risk of 10 + % absenteeism (54.8%); COH scores of >52.0 indicated lower risk (45.2%). CART item analysis identified one main subgroup at elevated risk for 10 + % absenteeism (node at 87.5% probability): (1) items 74 (true; hard to be by self without hurting feelings; IND) and 77 (false; members go out a lot; ARO). The tree-model’s accuracy in predicting 10 + % absenteeism was approximately 78.3%.

**Table 3:** FES subscale standard scores predictive of 10 + % absenteeism.

	Higher risk	Lower risk
Organization	53.0–58.0 (23.4%)	48.0–53.0 (2.5%)
Moral-religious emphasis	<=61.0 (5.2%)	61.0–65.9 (2.1%)
Expressiveness	>51.5 (7.3%)	46.8–51.5 (2.1%)
Intellectual-cultural orientation	47.0–58.0 (6.2%)	<35.9 (3.1%)
Achievement orientation	>53.0 (3.7%)	46.8–51.5 (2.6%)
Conflict	<=44.0 (2.2%)	>44.0 (2.1%)

*Subscales presented in descending order of impact*.

### Absenteeism: Higher Levels

CHAID analyses were also conducted on an exploratory basis for absenteeism levels of 15 + %, 20 + %, 30 + %, and 40 + %. The final collective tree-model that best differentiated youth with 15 + % absenteeism from youth with <15% absenteeism correctly identified 52.9% of participants and identified several items (14, 28, 42, 61, 71, and 75) and subscale scores as risk factors. The tree-model demonstrated higher specificity than sensitivity. MRE scores of >61.0 indicated higher risk of 15 + % absenteeism (17.0%); MRE scores of <= 43.9 indicated lower risk (10.9%). ACH scores of <=47 indicated higher risk of 15 + % absenteeism (16.6%); ACH scores of >59.0 indicated lower risk (5.4%). CTL scores of >47.2 indicated higher risk of 15 + % absenteeism (6.2%); CTL scores of 42.9–47.2 indicated lower risk (2.3%). IND scores of 51–53 indicated higher risk of 15 + % absenteeism (4.7%); IND scores of >53.0 indicated lower risk (2.6%). ARO scores of <=48.0 indicated higher risk of 15 + % absenteeism (3.3%); ARO scores of >48.0 indicated lower risk (2.6%). The final node representing highest overall risk of 15 + % absenteeism (0.867) included MRE scores of 56.0–61.0, item 42 (true; doing things spur of the moment; EXP), and item 75 (true; work before play is the rule; ICO). Independent analysis of predictors revealed that ACH scores significantly predicted 15 + % of days missed (*p* < 0.04, *F* = 8.16, predicted = 0.47). ACH scores of <=47.0 indicated higher risk of 15 + % absenteeism (52.2%); ACH scores of >47.0 indicated lower risk (47.8%).

The final collective tree-model that best differentiated youth with 20 + % absenteeism from youth with <20% absenteeism correctly identified 61.4% of participants and identified several items (4, 49, and 79) and subscale scores as risk factors. The tree-model demonstrated higher specificity than sensitivity. COH scores of 23.0–45.9 indicated higher risk of 20 + % absenteeism (27.9%); COH scores of >65.0 indicated lower risk (9.8%). CTL scores of 23.0–45.9 indicated higher risk of 20 + % absenteeism (27.9%); CTL scores of >65.0 indicated lower risk (9.8%). EXP scores of 34.0–47.0 indicated higher risk of 20 + % absenteeism (10.0%); EXP scores of <= 34.0 indicated lower risk (4.9%). MRE scores of >61 indicated higher risk of 20 + % absenteeism (5.1%); MRE scores of 43.9–51.0 indicated lower risk (2.4%).

The final collective tree-model that best differentiated youth with 30 + % absenteeism from youth with <30% absenteeism correctly identified 75.0% of participants and identified several items (18, 20, 30, 43, and 85) and subscale scores as risk factors. The tree-model demonstrated higher specificity than sensitivity. COH scores of 23.0–45.9 indicated higher risk of 30 + % absenteeism (27.9%); COH scores of 52–52.6 indicated lower risk (6.5%). MRE scores of 36.0–46.0 indicated higher risk of 30 + % absenteeism (4.0%); MRE scores of <=36 indicated lower risk (3.1%). EXP scores of 34.0–47.0 indicated higher risk of 30 + % absenteeism (10.0%); EXP scores of <= 34.0 indicated lower risk (4.9%). IND scores of >37.0 indicated higher risk of 30 + % absenteeism (7.2%); IND scores of <= 37.0 indicated lower risk (4.2%). CTL scores of <=43.0 indicated higher risk of 30 + % absenteeism (3.9%); CTL scores of >53.3 indicated lower risk (3.7%). CON scores of 44.0–54.3 indicated higher risk of 30 + % absenteeism (6.9%); CON scores of 38.5–43.0 indicated lower risk (2.4%). Independent analysis of the predictors revealed that ACH scores significantly predicted 30 + % of days missed (*p* < 0.05, *F* = 7.87). ACH scores of <=51.0 indicated higher risk of 30 + % absenteeism (52.5%); ACH scores of >51.0 indicated lower risk (47.5%).

The final collective tree-model that best differentiated youth with 40 + % absenteeism from youth with <40% absenteeism correctly identified 85.0% of participants and identified several items (10, 49, and 55) and subscale scores as risk factors. The tree-model demonstrated higher specificity than sensitivity. COH scores of 23.0–45.9 indicated higher risk of 40 + % absenteeism (10.2%); COH scores of 52.6–59 indicated lower risk (3.2%). MRE scores of 46.0–61.0 indicated higher risk of 40 + % absenteeism (38.8%); MRE scores of <=36 indicated lower risk (7.5%). ORG scores of <=53.0 indicated higher risk of 40 + % absenteeism (16.2%); ORG scores of >53.0 indicated lower risk (6.6%). IND scores of <=51 indicated higher risk of 40 + % absenteeism (5.2%); IND scores of >51.0 indicated lower risk (5.0%). ARO scores of <=61.0 indicated higher risk of 40 + % absenteeism (5.4%); ARO scores of >61.0 indicated lower risk (25.0%).

## Discussion

The present study examined family environment variables as potential predictors of various absenteeism severity levels. The findings reveal that several family environment variables are indeed related to different severity levels in both broad and more nuanced ways. Broadly, as expected, family environment risk factors tended to be more circumscribed and informative at higher levels of absenteeism, with much greater diversity at lower levels. Higher levels of absenteeism (i.e., 15 + %) appear more closely related to lower achievement orientation, active-recreational orientation, cohesion, and expressiveness. Lower levels of absenteeism (i.e., 1, 3, and 5%) were generally associated with a wider array of family environment variables.

Active-recreational standard scores were generally suppressed across absenteeism severity levels, a result that parallels Hansen et al.’s (1998) finding that less active families were associated with greater levels of school absenteeism among youth with anxiety-based conditions. These authors speculated that a low emphasis on social and physical activities and greater time spent at home may mean that some children may be more apt to spend school time at home. In addition, these children may be more predisposed to have difficulties with social skills and peer interactions that could also interfere with school attendance. Some have also found that school absenteeism is related to less participation in school sports (Hunt and Hopko, 2009), though others have not (Skedgell and Kearney, 2018). Lower active-recreational scores were evident as well in Kearney and Silverman’s (1995) study that led those authors to conclude that some families of youth with absentee problems are isolated in nature.

A number of nuanced findings were also revealed in the present study, however, that deserve detailed description. With respect to achievement orientation, for example, elevated standard scores were associated with less absenteeism severity but lower standard scores were associated with greater absenteeism severity. Higher school performance is generally associated with higher competition (Harrison and Rouse, 2014), though effects can depend on gender and age (Little and Garber, 2004; Wang and Holcombe, 2010). At the family level, achievement orientation could translate into specific activities such as modeling academic advancement, reading frequently, encouraging a strong work ethic, and providing enrichment opportunities that distally affect school attendance (Dubow et al., 2009).

In addition, lower standard scores for expressiveness were evident at less severe (3, 5%) and more severe (20, 30%) levels of absenteeism, though elevated standard scores were predictive of 10 + % absenteeism. As noted earlier, Bernstein and Borchardt (1996) found that families of youth with school refusal displayed significant problems with respect to role performance and communication. Findings from the present study indicate that such difficulties may be less evident during periods when families are working together to solve an absentee problem and during periods when frustration over long-term absenteeism has led to greater disengagement and less opportunities for direct expression (Kearney and Silverman, 1995).

Family cohesion represented another nuanced finding. Cohesion was not predictive at 1 + % and 3 + % absenteeism but lower standard scores were more predictive of higher levels of absenteeism. This result parallels Bernstein et al.’s (1999) finding that adolescents with school attendance problems and their parents viewed their families as particularly rigid and disengaged on a cohesion dimension. In addition, several researchers have found, broadly speaking, that parent and family involvement and support are crucial variables with respect to school attendance, performance, and dropout (Sheldon, 2007; Topor et al., 2010; Parr and Bonitz, 2015). Cohesion in the form of help with homework, support for academic progress, and commitment to education may be a key in this regard (Wilder, 2014).

Family conflict was expected to be an important predictor of absenteeism severity in the present study. Elevated conflict standard scores were more predictive of 5 + % absenteeism severity, whereas lower conflict standard scores were more predictive of 10 + % absenteeism severity. Some have found family conflict to be elevated in this population in general, and advocate for the problem to be resolved clinically in this population (Kearney and Silverman, 1995; Kearney and Albano, 2018), though others have found family conflict to be unrelated to school attendance problems (McShane et al., 2001). As with expressiveness, some families may display increased conflict at a point of urgency when trying to resolve a school attendance problem but later become frustrated and disengaged from the process (Kearney, 2019).

Finally, control was a family environment variable that did not appear until higher levels of absenteeism severity. Lower levels of control were more predictive at higher levels of absenteeism severity, particularly at the 20 + % and 30 + % levels. A less structured home environment has been associated with school absenteeism in other studies (Hunt and Hopko, 2009). In addition, as mentioned earlier, Bernstein et al. (1990) found that inconsistency of family rules related to some youth with school attendance problems. Conversely, family rules are part of a parent involvement process often associated with academic success (Catsambis, 2001).

Analyses of individual FES items also revealed interesting findings. First, items were sometimes endorsed differently in different nodes, indicating a high level of variability in these groups. This applied particularly to lower levels of absenteeism. Second, fewer items were predictive of 10 + % absenteeism than at lower levels, mirroring the subscale finding that predictors tended to be more restricted at higher absenteeism severity levels. Overall, however, examining subscale scores appeared to be more useful than examining item scores.

The present study may thus have some applicability to MTSS models of school absenteeism and how tiers within these models may be demarcated. In particular, absenteeism severity levels of 10–15% appear to be associated with more defined sets of risk factors, which may indicate more qualitative changes in family functioning at these levels. More intense drops in achievement orientation, active-recreational orientation, cohesion, and expressiveness, in addition to less conflict, may indicate that families become substantially more disengaged at these levels. Such disengagement could come in the form of sharply reduced parent-school official contact, consequences for school absenteeism, academic assistance, attendance monitoring, and parent supervision (Kearney and Albano, 2018).

The results may also have implications for MTSS development in educational settings. Many local educational agencies, for example, are moving toward systemic, evidence-based systems of academic and behavioral supports to meet the unique needs of diverse students (McIntosh and Goodman, 2016). A better understanding of how these needs intersect with family-based challenges is essential in this respect. Parental involvement, for example, has been found to be a key element of success in MTSS programs, and such programs often benefit from a wider array of stakeholders that include parents (August et al., 2018). In addition, MTSS models are increasingly moving toward a “whole child” approach that more fully considers ecological levels outside of school, such as family factors (Sailor et al., 2018). Results of the present study and related studies may thus help inform such an approach.

Results of the present study also have implications for further research work in this area, particularly with respect to how these findings intersect with other family-based risk factors for school absenteeism. Gubbels et al. (2019), for example, conducted a meta-analytic review of such factors for school absenteeism and dropout and found several pertinent family domains. These included low parental school involvement, lack of nuclear family structure, and low parental control, among others. An understanding of how the family environment dynamics identified in the present study intersect with these broader domains, particularly with respect to specific levels of school absenteeism, would be quite instructive for subtyping and demarcation purposes. Such information may also help inform family-based treatment for this population. For example, Tobias (2019) found that family-based intervention for persistent school absenteeism was often hindered by an insecure home environment. The latter construct could be investigated in greater detail in future work to identify whether the dynamics noted in the present study would apply.

Limitations of the present study should be noted. First, the sample was a diverse one ranging from having no formal school absences to having many school absences. Second, more detailed analyses of absenteeism type or of demographic or developmental differences were not examined in accordance with sample constraints and diversity of settings. Third, the primary dependent measure was based on parent-report. Future researchers should endeavor to explore a more wide-ranging assessment of family functioning in this population.

## Conclusion

Despite these limitations, findings from the present study may have some clinical implications. Educators, mental health professionals, and others who address these families, particularly at higher levels of absenteeism severity, will likely need to prioritize certain goals given the problematic family dynamics involved. With respect to school attendance, such goals may include repairing parent-school official communications, educating family members about creative educational options, and establishing contracts or agreements to improve problem-solving ability and increase incentives for attending school (Kearney, 2019). More broadly, such goals may include interventions to enhance family engagement and communication as well as contacts with outside sources of support (Kelly et al., 2018).

## Data Availability Statement

The datasets generated for this study are available on request to the corresponding author.

## Ethics Statement

The studies involving human participants were reviewed and approved by UNLV IRB. Written informed consent to participate in this study was provided by the participants’ legal guardian/next of kin.

## Author Contributions

All authors revised and approved the submitted version. MF helped collect data, performed the initial analyses, and assisted in the writing of the manuscript. CK helped with data analysis, assisted in the writing of the manuscript, and supervised the study.

### Conflict of Interest

The authors declare that the research was conducted in the absence of any commercial or financial relationships that could be construed as a potential conflict of interest.

## References

[ref1] Attwood G. Croll P. (2015). Truancy and well-being among secondary school pupils in England. Educ. Stud. 41, 14–28. 10.1080/03055698.2014.955725

[ref2] August G. J. Piehler T. F. Miller F. G. (2018). Getting “SMART” about implementing multi-tiered systems of support to promote school mental health. J. Sch. Psychol. 66, 85–96. 10.1016/j.jsp.2017.10.001 29429498 PMC5812372

[ref3] Barber B. K. Buehler C. (1996). Family cohesion and enmeshment: different constructs, different effects. J. Marriage Fam. 58, 433–441. 10.2307/353507

[ref4] Berk R. A. (2006). An introduction to ensemble methods for data analysis. Soc. Methods Res. 34, 263–295. 10.1177/0049124105283119

[ref5] Bernstein G. A. Borchardt C. M. (1996). School refusal: family constellation and family functioning. J. Anxiety Disord. 10, 1–19. 10.1016/0887-6185(95)00031-3

[ref6] Bernstein G. A. Garfinkel B. D. Borchardt C. M. (1990). Comparative studies of pharmacotherapy for school refusal. J. Am. Acad. Child Adolesc. Psychiatry 29, 773–781. 10.1097/00004583-199009000-00016 2228932

[ref7] Bernstein G. A. Warren S. L. Massie E. D. Thuras P. D. (1999). Family dimensions in anxious-depressed school refusers. J. Anxiety Disord. 13, 513–528. 10.1016/S0887-6185(99)00021-3, PMID: 10600052

[ref8] Berryhill M. B. Hayes A. Lloyd K. (2018). Chaotic-enmeshment and anxiety: the mediating role of psychological flexibility and self-compassion. Contemp. Fam. Ther. 40, 326–337. 10.1007/s10591-018-9461-2

[ref9] Bradley R. H. Barrett K. W. Conners-Burrow N. A. McKelvey L. M. Whiteside-Mansell L. Casey P. H. (2010). Growing up in violent communities: do family conflict and gender moderate impacts on adolescents’ psychosocial development? J. Abnorm. Child Psychol. 39, 95–107. 10.1007/s10802-010-9448-4 20694576

[ref10] Bridgeland J. M. DiIulio J. J. Jr. Morison K. B. (2006). The silent epidemic: Perspectives of high school dropouts. Civic enterprises. Washington, D.C.: Civic Enterprises. Available at: https://docs.gatesfoundation.org/documents/thesilentepidemic3-06final.pdf (Accessed June 14, 2019).

[ref11] Burrus J. Roberts R. D. (2012). Dropping out of high school: Prevalence, risk factors, and remediation strategies. Princeton, NJ: R&D Connections Available at: https://www.researchgate.net/profile/Richard_Roberts2/publication/233381528_Dropping-out_of_High_School_Prevalence_risk_factors_and_remediation_strategies/links/09e41509e7ca9975a7000000.pdf (Accessed June 14, 2019).

[ref12] Burton C. M. Marshal M. P. Chisolm D. J. (2014). School absenteeism and mental health among sexual minority youth and heterosexual youth. J. Sch. Psychol. 52, 37–47. 10.1016/j.jsp.2013.12.001, PMID: 24495493 PMC4058829

[ref13] Catsambis S. (2001). Expanding knowledge of parental involvement in children’s secondary education: connections with high school seniors’ academic success. Soc. Psychol. Educ. 5, 149–177. 10.1023/A:1014478001512

[ref14] Chen I. J. Wu Y. Wei B. S. (2017). Effects of anxious emotions on the behavioral problems of Chinese children with learning difficulties. Qual. Quant. 51, 1147–1165. 10.1007/s11135-016-0322-5

[ref15] Christenson S. L. Thurlow M. L. (2004). School dropouts: prevention considerations, interventions, and challenges. Curr. Dir. Psychol. Sci. 13, 36–39. 10.1111/j.0963-7214.2004.01301010.x

[ref16] Chu B. C. Guarino D. Mele C. O’Connell J. Coto P. (2018). Developing an online early detection system for school attendance problems: results from a research-community partnership. Cogn. Behav. Pract. 26, 35–45. 10.1016/j.cbpra.2018.09.001

[ref17] Chung J. Y. Lee S. (2019). Dropout early warning systems for high school students using machine learning. Child Youth Serv. Rev. 96, 346–353. 10.1016/j.childyouth.2018.11.030

[ref18] Coffey S. E. Stallworth L. Majors T. Higgs K. Gloster L. Carter Y. (2018). Using the cluster support team and multi-tiered systems of support to provide wraparound services in a large urban school district. Savannah, GA: National Youth-At-Risk Conference, Georgia Southern University Available at: https://digitalcommons.georgiasouthern.edu/nyar_savannah/2018/2018/18 (Accessed June 14, 2019).

[ref19] Conry J. M. Richards M. P. (2018). The severity of state truancy policies and chronic absenteeism. J. Educ. Stud. Placed Risk 23, 187–203. 10.1080/10824669.2018.1439752

[ref20] Cordero J. M. Santín D. Simancas R. (2017). Assessing European primary school performance through a conditional nonparametric model. J. Oper. Res. Soc. 68, 364–376. 10.1057/jors.2015.42

[ref500] Dahl P. (2016). Factors associated with truancy: Emerging adults’ recollections of skipping school. J. Adolesc. Res. 31, 119–138. 10.1177/0743558415587324

[ref21] Davies P. T. Cummings E. M. Winter M. A. (2004). Pathways between profiles of family functioning, child security in the interparental subsystem, and child psychological problems. Dev. Psychopathol. 16, 525–550. 10.1017/S0954579404004651, PMID: 15605624

[ref22] De Witte K. Cabus S. Thyssen G. Groot W. Van Den Brink H. M. (2013). A critical review of the literature on school dropout. Educ. Res. Rev. 10, 13–28. 10.1016/j.edurev.2013.05.002

[ref23] DePaoli J. L. Fox J. H. Ingram E. S. Maushard M. Bridgeland J. M. Balfanz R. (2015). Building a grad nation: Progress and challenge in ending the high school dropout epidemic. Annual update. Washington, D.C: Civic Enterprises, 2015 Available at: https://www.americaspromise.org/building-grad-nation-report (Accessed June 14, 2019).

[ref24] Dubow E. F. Boxer P. Huesmann L. R. (2009). Long-term effects of parents’ education on children’s educational and occupational success. Merrill-Palmer Q. 55, 224–249. 10.1353/mpq.0.0030 PMC2853053 20390050

[ref25] Eagle J. W. Dowd-Eagle S. E. Snyder A. Holtzman E. G. (2015). Implementing a multi-tiered system of support (MTSS): collaboration between school psychologists and administrators to promote systems-level change. J. Educ. Psychol. Consult. 25, 160–177. 10.1080/10474412.2014.929960

[ref26] Elliott J. G. Place M. (2019). School refusal: developments in conceptualisation and treatment since 2000. J. Child Psychol. Psychiatry 60, 4–15. 10.1111/jcpp.12848, PMID: 29197106

[ref27] Epstein J. L. Sheldon S. B. (2002). Present and accounted for: improving student attendance through family and community involvement. J. Educ. Res. 95, 308–318. 10.1080/00220670209596604

[ref28] Freeman J. Simonsen B. (2015). Examining the impact of policy and practice interventions on high school dropout and school completion rates: a systematic review of the literature. Rev. Educ. Res. 85, 205–248. 10.3102/0034654314554431

[ref29] Freeman J. Simonsen B. McCoach D. B. Sugai G. Lombardi A. Horner R. (2016). Relationship between school-wide positive behavior interventions and supports and academic, attendance, and behavior outcomes in high schools. J. Posit. Behav. Interv. 18, 41–51. 10.1177/1098300715580992

[ref30] Gentle-Genitty C. Karikari I. Chen H. Wilka E. Kim J. (2015). Truancy: a look at definitions in the USA and other territories. Educ. Stud. 41, 62–90. 10.1080/03055698.2014.955734

[ref31] Gubbels J. van der Put C. E. Assink M. (2019). Risk factors for school absenteeism and dropout: a meta-analytic review. J. Youth Adolesc. 48, 1637–1667. 10.1007/s10964-019-01072-5, PMID: 31312979 PMC6732159

[ref32] Hansen C. Sanders S. L. Massaro S. Last C. G. (1998). Predictors of severity of absenteeism in children with anxiety-based school refusal. J. Clin. Child Adolesc. Psychol. 27, 246–254. 10.1207/s15374424jccp2703_2 9789185

[ref33] Harrison J. Rouse P. (2014). Competition and public high school performance. Socio-Econ. Plan. Sci. 48, 10–19. 10.1016/j.seps.2013.11.002

[ref34] Havik T. Bru E. Ertesvåg S. K. (2015). School factors associated with school refusal-and truancy-related reasons for school non-attendance. Soc. Psychol. Educ. 18, 221–240. 10.1007/s11218-015-9293-y

[ref35] Hill N. E. Taylor L. C. (2004). Parental school involvement and children’s academic achievement: pragmatics and issues. Curr. Dir. Psychol. Sci. 13, 161–164. 10.1111/j.0963-7214.2004.00298.x

[ref36] Hunt M. K. Hopko D. R. (2009). Predicting high school truancy among students in the Appalachian South. J. Prim. Prev. 30, 549–567. 10.1007/s10935-009-0187-7, PMID: 19680814

[ref37] Ingul J. M. Havik T. Heyne D. (2019). Emerging school refusal: a school-based framework for identifying early signs and risk factors. Cogn. Behav. Pract. 26, 46–62. 10.1016/j.cbpra.2018.03.005

[ref38] Ingul J. M. Klöckner C. A. Silverman W. K. Nordahl H. M. (2012). Adolescent school absenteeism: modelling social and individual risk factors. Child Adolesc. Ment. Health 17, 93–100. 10.1111/j.1475-3588.2011.00615.x 32847296

[ref39] Jaycox L. H. Repetti R. L. (1993). Conflict in families and the psychological adjustment of preadolescent children. J. Fam. Psychol. 7, 344–355. 10.1037/0893-3200.7.3.344

[ref40] Kearney C. A. (2008). An interdisciplinary model of school absenteeism in youth to inform professional practice and public policy. Educ. Psychol. Rev. 20, 257–282. 10.1007/s10648-008-9078-3

[ref41] Kearney C. A. (2016). Managing school absenteeism at multiple tiers: An evidence-based and practical guide for professionals. Oxford, UK: Oxford University Press.

[ref42] Kearney C. A. (2019). Helping families of youth with school attendance problems: A practical guide for mental health and school-based professionals. New York: Oxford University Press.

[ref43] Kearney C. A. Albano A. M. (2018). When children refuse school: Therapist guide. New York: Oxford University Press.

[ref44] Kearney C. A. Graczyk P. (2014). A response to intervention model to promote school attendance and decrease school absenteeism. Child Youth Care Forum 43, 1–25. 10.1007/s10566-013-9222-1

[ref45] Kearney C. A. Silverman W. K. (1995). Family environment of youngsters with school refusal behavior: a synopsis with implications for assessment and treatment. Am. J. Fam. Ther. 23, 59–72. 10.1080/01926189508251336

[ref46] Kelly J. Rossen E. Cowan K. C. (2018). Supporting families to support students. Educ. Leadersh. 75, 62–69. 10.4324/9780203417003-13

[ref47] Lagana M. T. (2004). Protective factors for inner-city adolescents at risk of school dropout: family factors and social support. Child. Sch. 26, 211–220. 10.1093/cs/26.4.211

[ref48] Lahti M. Evans C. B. Goodman G. Schmidt M. C. LeCroy C. W. (2019). Parents as teachers (PAT) home-visiting intervention: a path to improved academic outcomes, school behavior, and parenting skills. Child Youth Serv. Rev. 99, 451–460. 10.1016/j.childyouth.2019.01.022

[ref49] Lemon S. Roy J. Clark M. Friedmann P. Rakowski W. (2003). Classification and regression tree analysis in public health: methodological review and comparison with logistic regression. Ann. Behav. Med. 26, 172–181. 10.1207/S15324796ABM2603_02, PMID: 14644693

[ref50] Lindblom J. Peltola M. J. Vänskä M. Hietanen J. K. Laakso A. Tiitinen A. (2017). Early family system types predict children’s emotional attention biases at school age. Int. J. Behav. Dev. 41, 245–256. 10.1177/0165025415620856

[ref51] Little S. A. Garber J. (2004). Interpersonal and achievement orientations and specific stressors predict depressive and aggressive symptoms. Int. J. Adolesc. Youth 19, 63–84. 10.1177/0743558403258121

[ref52] Makihara H. Nagaya M. Nakajima M. (1985). An investigation of neurotic school refusal in one-parent families. Jpm. J. Child Adols. Psy. 26, 303–315.

[ref53] Maxwell L. E. (2016). School building condition, social climate, student attendance and academic achievement: a mediation model. J. Environ. Psychol. 46, 206–216. 10.1016/j.jenvp.2016.04.009

[ref54] Mazerolle L. Eggins E. Sydes M. Hine L. McEwan J. Norrie G. (2018). Criminal justice responses to domestic and family violence: A rapid review of the evaluation literature. University of Queensland, Australia: Domestic and Family Violence Review Unit Available at: https://www.courts.qld.gov.au/__data/assets/pdf_file/0006/586185/systematic-review-of-criminal-justice-responses-to-domestic-and-family-violence.pdf (Accessed June 14, 2019).

[ref55] McIntosh K. Goodman S. (2016). Integrated multi-tiered systems of support: Blending RTI and PBIS. New York: Guilford.

[ref56] McKee M. T. Caldarella P. (2016). Middle school predictors of high school performance: a case study of dropout risk indicators. Education 136, 515–529.

[ref57] McShane G. Walter G. Rey J. M. (2001). Characteristics of adolescents with school refusal. Aust. N. Z. J. Psychiatry 35, 822–826. 10.1046/J.1440-1614.2001.00955.X 11990893

[ref58] Moos R. H. (1990). Conceptual and empirical approaches to developing family-based assessment procedures: resolving the case of the family environment scale. Fam. Process 29, 199–208; discussion 209–211. 10.1111/j.1545-5300.1990.00199.x, PMID: 2373215

[ref59] Moos R. H. Moos B. S. (2009). Family environment scale manual and sampler set: Development, applications and research. Palo Alto, CA: Mind Garden, Inc.

[ref60] Parr A. K. Bonitz V. S. (2015). Role of family background, student behaviors, and school-related beliefs in predicting high school dropout. Am. Educ. Res. J. 108, 504–514. 10.1080/00220671.2014.917256

[ref61] Pullen P. C. Kennedy M. J. (Eds.) (2019). Handbook of response to intervention and multi-tiered systems of support. New York: Routledge.

[ref62] Ready P. D. (2010). Leishmaniasis emergence in Europe. Eur. Secur. 15:19505. 10.1016/s0140-6736(00)62841-6, PMID: 20403308

[ref63] Rocque M. Jennings W. G. Pquero A. R. Ozkan T. Farrington D. P. (2017). The importance of school attendance: findings from the Cambridge study in delinquent development on the life-course effects of truancy. J. Res. Crime Delinq. 63, 592–612. 10.1177/0011128716660520

[ref64] Rosellini A. J. Dussaillant F. Zubizarreta J. R. Kessler R. C. Rose S. (2018). Predicting posttraumatic stress disorder following a natural disaster. J. Psychiatr. Res. 96, 15–22. 10.1016/j.jpsychires.2017.09.010, PMID: 28950110 PMC5726547

[ref65] Sailor W. McCart A. B. Choi J. H. (2018). Reconceptualizing inclusive education through multi-tiered system of support. Inc 6, 3–18. 10.1352/2326-6988-6.1.3

[ref66] Sansone D. (2019). Teacher characteristics, student beliefs, and the gender gap in STEM fields. Educ. Eval. Policy Anal. 41, 127–144. 10.3102/0162373718819830

[ref67] Sheldon S. B. (2007). Improving student attendance with school, family, and community partnerships. J. Educ. Res. 100, 267–275. 10.3200/JOER.100.5.267-275

[ref68] Skedgell K. K. Kearney C. A. (2016). Predictors of absenteeism severity in truant youth: a dimensional and categorical analysis. Am. Second. Educ. 45, 46–58. 10.1016/j.childyouth.2018.01.043

[ref69] Skedgell K. K. Kearney C. A. (2018). Predictors of school absenteeism severity at multiple levels: a classification and regression tree analysis. Child Youth Serv. Rev. 86, 236–245. 10.1016/j.childyouth.2018.01.043

[ref70] Spruyt B. Keppens G. Kemper R. Bradt L. (2016). If only they had a file on every pupil’: on the mismatch between truancy policy and practice in Flanders. Int. J. Soc. Educ. 26, 171–189. 10.1080/09620214.2016.1191965

[ref71] Tobias A. (2019). A grounded theory study of family coach intervention with persistent school non-attenders. Educ. Psychol. Pract. 35, 17–33. 10.1080/02667363.2018.1518215

[ref72] Topor D. R. Keane S. P. Shelton T. L. Calkins S. D. (2010). Parent involvement and student academic performance: a multiple mediational analysis. J. Prev. Interv. Community 38, 183–197. 10.1080/10852352.2010.486297, PMID: 20603757 PMC3020099

[ref73] Tucker M. C. Rodriguez C. M. (2014). Family dysfunction and social isolation as moderators between stress and child physical abuse risk. J. Fam. Violence 29, 175–186. 10.1007/s10896-013-9567-0

[ref74] UNESCO (2012). Education data, UNESCO institute of statistics. Retrieved March 6, 2019. Available at: http://www.uis.unesco.org/Education/ (Accessed June 14, 2019).

[ref75] Wahler R. G. (1980). The insular mother: her problems in parent-child treatment. J. Appl. Behav. Anal. 13, 207–219. 10.1901/jaba.1980.13-207, PMID: 7380748 PMC1308126

[ref76] Wang M. T. Holcombe R. (2010). Adolescents’ perceptions of school environment, engagement, and academic achievement in middle school. Am. Educ. Res. J. 47, 633–662. 10.3102/0002831209361209

[ref77] Weiss M. Cain B. (1964). The residential treatment of children and adolescents with school phobia. Am. J. Orthopsychiatry 34, 103–114. 10.1111/j.1939-0025.1964.tb02198.x, PMID: 14111399

[ref78] Wilder S. (2014). Effects of parental involvement on academic achievement: a meta-synthesis. Educ. Rev. 66, 377–397. 10.1080/00131911.2013.780009

